# Genome sequencing identified novel mechanisms underlying virescent mutation in upland cotton *Gossypiuma hirsutum*

**DOI:** 10.1186/s12864-021-07810-z

**Published:** 2021-07-03

**Authors:** Jin Gao, Yang Shi, Wei Wang, Yong-Hui Wang, Hua Yang, Qing-Hua Shi, Jian-Ping Chen, Yan-Ru Sun, Li-Wang Cai

**Affiliations:** Jiangsu Coastal Area Institute of Agricultural Sciences/Observation and Experimental Station of Saline Land of Costal Area, Ministry of Agriculture, Yancheng, 224002 Jiangsu People’s Republic of China

**Keywords:** Virescent mutant, Sumian 22, *ABCI1*, Rapid mapping, Genome, Chlorophyll synthesis

## Abstract

**Background:**

Virescent mutation broadly exists in plants and is an ideal experimental material to investigate regulatory mechanisms underlying chlorophyll synthesis, photosynthesis and plant growth. Up to date, the molecular mechanisms in two virescent mutations have been clarified in cottons (*Gossypiuma hirsutum*). A virescent mutation has been found in the cotton strain Sumian 22, and the underlying molecular mechanisms have been studied.

**Methods:**

The virescent mutant and wild type (WT) of Sumian 22 were cross-bred, and the F_1_ population were self-pollinated to calculate the segregation ratio. Green and yellow leaves from F_2_ populations were subjected to genome sequencing and bulked-segregant analysis was performed to screen mutations. Real-time quantitative PCR (RT-qPCR) were performed to identify genes in relations to chlorophyll synthesis. Intermediate products for chlorophyll synthesis were determined to validate the RT-qPCR results.

**Results:**

The segregation ratio of green and virescent plants in F2 population complied with 3:1. Compared with WT, a 0.34 Mb highly mutated interval was identified on the chromosome D10 in mutant, which contained 31 genes. Among them, only *ABCI1* displayed significantly lower levels in mutant than in WT. Meanwhile, the contents of Mg-protoporphyrin IX, protochlorophyllide, chlorophyll a and b were all significantly lower in mutant than in WT, which were consistent with the inhibited levels of *ABCI1*. In addition, a mutation from A to T at the -317 bp position from the start codon of *ABCI1* was observed in the genome sequence of mutant.

**Conclusions:**

Inhibited transcription of *ABCI1* might be the mechanism causing virescent mutation in Sumian 22 cotton, which reduced the transportation of protoporphyrin IX to plastid, and then inhibited Mg-protoporphyrin IX, Protochlorophyllide and finally chlorophyll synthesis. These results provided novel insights into the molecular mechanisms underlying virescent mutation in cotton.

**Supplementary Information:**

The online version contains supplementary material available at 10.1186/s12864-021-07810-z.

## Background

As one of the important economic crops in the world, cotton is of great significance to provide materials for the global textile industry. Scientists are always trying to breed high-quality cotton breeds. Investigation of the molecular mechanisms underlying plant growth and production of cotton may facilitate the molecular breeding process.

Virescent mutation is characterized by yellowish leaves at the early stage, which gradually become normal green leaves at maturity [1]. The virescent mutation is an easily identifiable character and has been observed in various plant species including rape [2], *Arabidopsis* [3], rice [4], cucumber [5], maize [6] and cotton [7]. The virescent mutants in leaves directly or indirectly affect the biosynthesis pathway of chlorophyll, resulting in the imbalance of the content and proportion of photosynthetic pigments, finally changing the leaf color [8–10]. In general, the virescent mutation is genetically stable and the genetic mode of virescent mutant is simple, controlled by 1–2 pairs of recessive genes. Thus, virescent mutant plants together with wild type (WT) plants provide ideal experimental materials for investigations of mechanisms regulating the expression of genes in relation to chlorophyll synthesis, which are important to photosynthesis and plant growth.

In allotetraploid cotton strains, 22 virescent mutants have been identified, which were suspected to the mutations of 24 genes. However, to the best of our knowledge, only two molecular mechanisms have been clarified. First, in the virescent mutant of the cotton strain T582, the normal functioning of Mg-chelatase I subunit (CHLI) was disturbed [11]. Next, this mutant was localized to a 20 kb interval on the chromosome 20 and the responsible gene was named *GhRVL*, which was homologous to *CHLI* in Arabidopsis [12]. Similar results were also found in the virescent phenotype of the cotton strain ZM050400 [1]. Compared with the cotton reference genome of the TM-1 stain [13], *GhRVL* was localized to the interval between 0.7 and 3.9 Mb on chromosome D10 in the TM-1 genome. Second, another virescent gene was identified on the chromosome D04 and the candidate gene was named *GhPUR4*, which affected the normal function of formylglycinamide ribotide amidotransferase (FGAMS) in the fourth step of the de novo purine biosynthesis pathway and finally resulted in the reduction of chlorophyll content, abnormal chloroplast development and virescent true leaves [14]. These results revealed the molecular mechanisms underlying virescent leaves in certain cotton strains. However, other virescent strains may not all follow these mechanisms. More studies are still required to further investigate the molecular mechanisms underlying virescent mutations in cotton.

Sumian 22 (*Gossypiuma hirsutum*) is an important upland cotton variety in China. It shows high yield, good quality of fiber, and high resistance to diseases [15]. In 2004, a natural virescent mutant was found in the Sumian 22 populations, whose leaves were yellow at the young stage but then turned to light green at the boll stage [16]. Clarifying the genetic basis of leaf color mutation has important theoretical and practical value in cotton research [17]. Considering the insufficient understanding of molecular mechanisms underlying virescent mutation in cotton, it is still necessary to also study the mechanisms underlying the virescent Sumian 22 strain.

Bulked-segregant analysis (BSA) is a very useful approach to identify genetic locus for simple quality trait, which separately pools the DNA of extreme individuals with contrasting phenotypes from a segregating population followed by screening of mutated molecular markers between parents and bulks [18, 19]. Based on next-generation sequencing (NGS), BSA analysis greatly accelerates the period of gene mapping research [20]. This method has been successfully applied to identify genes regulating genic male sterility in sesame [21], virescent mutation in rapeseed [22], plant height in maize [23] and rate of leaf initiation in barley [24].

In the present study, the virescent mutant (mutant) and wild green type (WT) of Sumian 22 were cross-bred and the F_2_ population was separated to green and yellow pools. BSA was conducted to search single nucleotide polymorphisms (SNP) between the two pools. Based on the SNP results, candidate genes were predicted, their mRNA levels were examined using real-time quantitative PCR (RT-qPCR) and the changes of potential intermediate metabolites for chlorophyll synthesis were also determined. Overall, these results aimed to explain the molecular mechanisms underlying the virescent trait in Sumian 22 cotton, which provides a basis to further understand the molecular mechanisms underlying the virescent phenomenon and regulation of chlorophyll synthesis in cotton.

## Results

### The virescent mutation is a recessive gene in Sumian 22

**Fig. 1:**
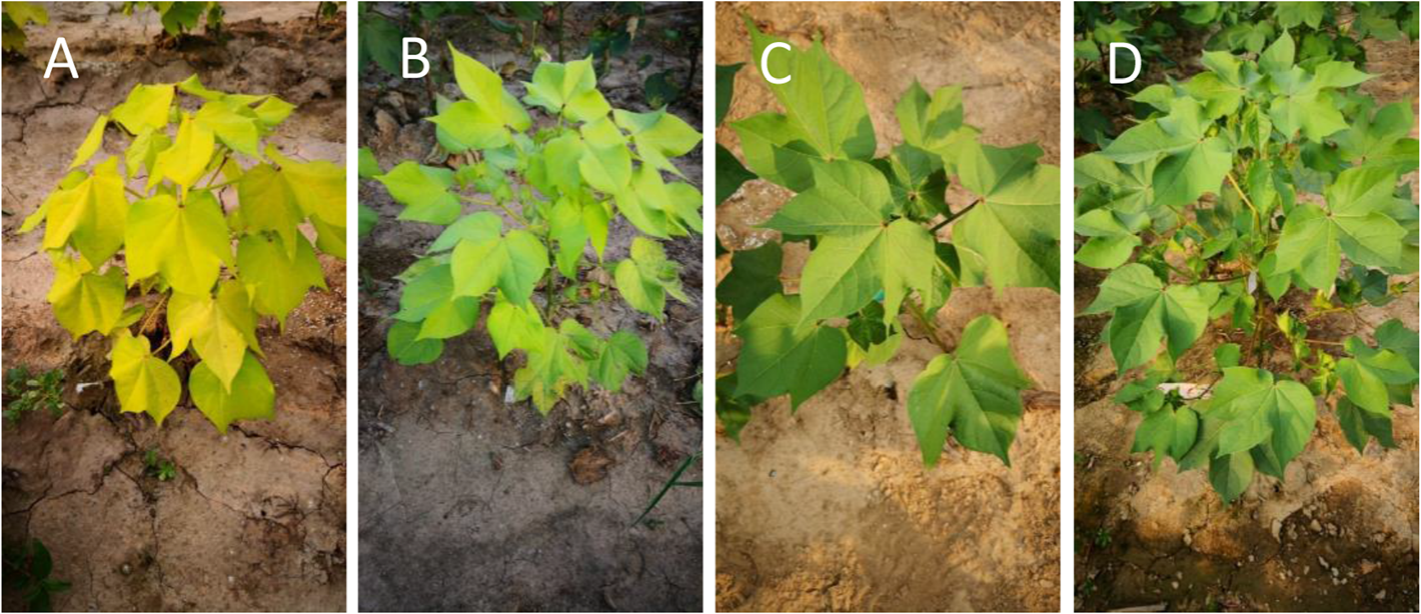
Phenotypes of leaves in the virescent mutant (Mutant) of Sumian 22 and wild type (WT) of Sumian 22. **A** Mutant at the young stage; **B** WT at the young stage; **C** mutant at maturity; **D** WT at maturity

The young leaves of mutant were clearly distinguished by a yellowish leaf color (Fig. 1A), significantly different from the WT (Fig. 1C). At the mature stage, mutant leaves became green (Fig. 1B), but still slightly different from the WT (Fig. 1D).

In the cross-breed between mutant and WT, all F_1_ plants revealed green leaves. However, in the self-pollinated F_2_ population, 72.8% and 27.2% individuals revealed green and yellow leaves, respectively. The ratio between green- and yellow-leaved plants was not significantly different from 3:1 (Table 1), suggesting the virescent mutation in Sumian 22 is a recessive mutation.

**Table 1:** Segregation of leaf color in the F_2_ population of the cross-breed between the wild type and the virescent mutant of Sumian 22 cotton

Cross	Wild type S22 × Virescent mutant S22
Number of green leaves plants	704
Number of yellow leaves plants	263
Total number of plants	967
Expected ratio	3:1
χ^2^	2.49

χ^2^_0.05_ = 3.84, χ^2^ < χ^2^_0.05_ indicates no significant difference between the observed and expected ratios

### Rapid delimitation of a candidate genomic region by BSA-seq

Genome sequencing obtained 124.46 and 127.67 million reads for WT and mutant, with the genome coverage of 94.66% and 94.63%, respectively. Considering the heterogeneity of plants in F_2_ population, the G- and Y-pools were sequenced, which produced 822.93 and 680.68 M reads, covering 95.30% and 95.30% of the whole genome, respectively (Additional file 1: Table S1).

**Fig. 2:**
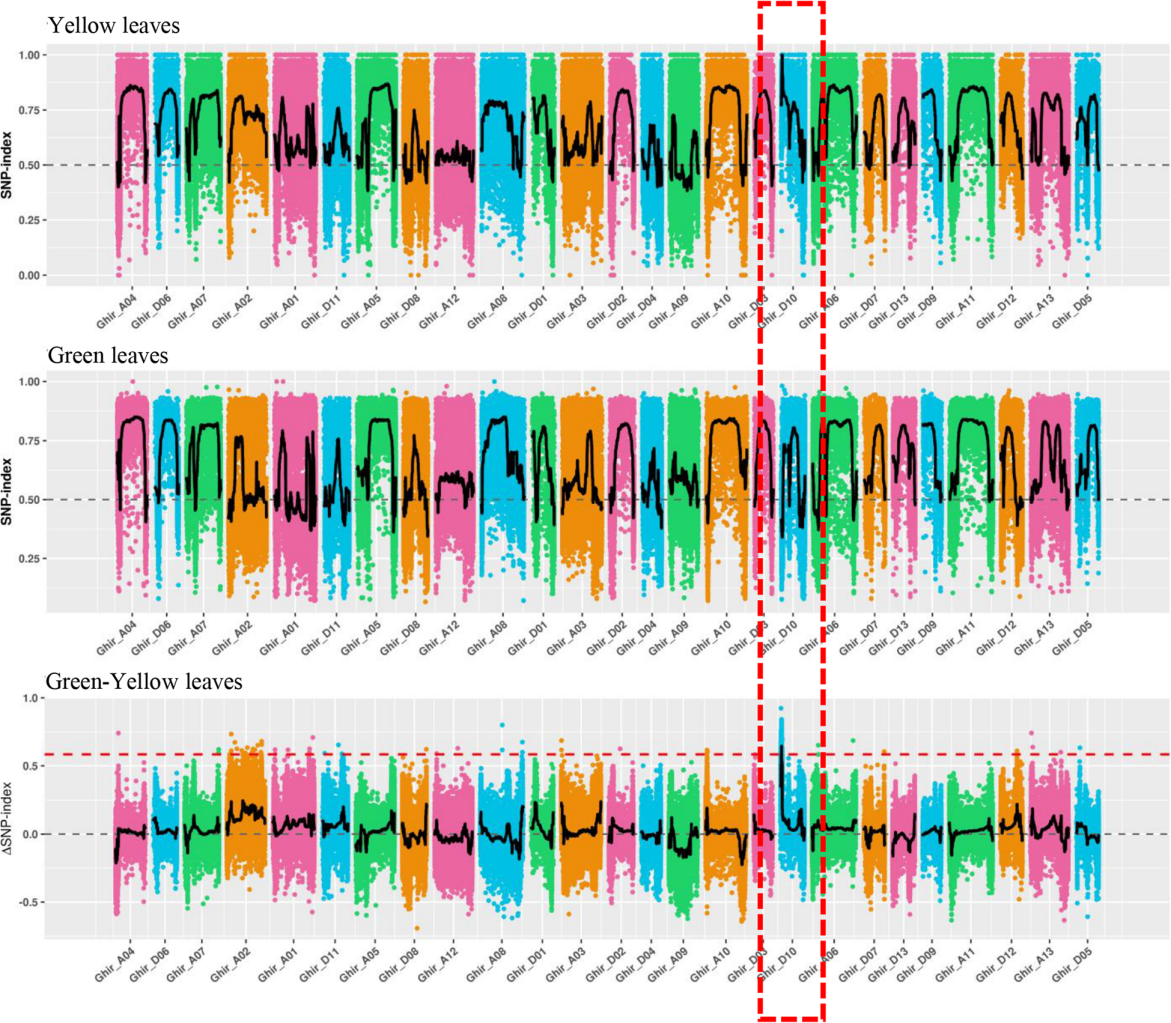
SNP index plot of green and yellow leaves (G-pool and Y-pool) in F_2_ population of the cross-breed between wild type (WT) and virescent mutant of Sumian 22

Between WT and mutant, 958,198 SNPs and 528,693 small InDels polymorphic markers were identified. The SNP-index plots were similar between G-pool and Y-pool at most regions of the genome. However, ΔSNP-index revealed a higher SNP load at the region from 3.08 to 3.42 Mb on the chromosome D10 according to the reference genome of TM-1 cotton [13] (Fig. 2), which was considered as the unique candidate region of the virescent gene. In this region, 31 coding genes and 597 SNPs were identified.

### Determination of gene transcription levels by RT-qPCR

**Fig. 3:**
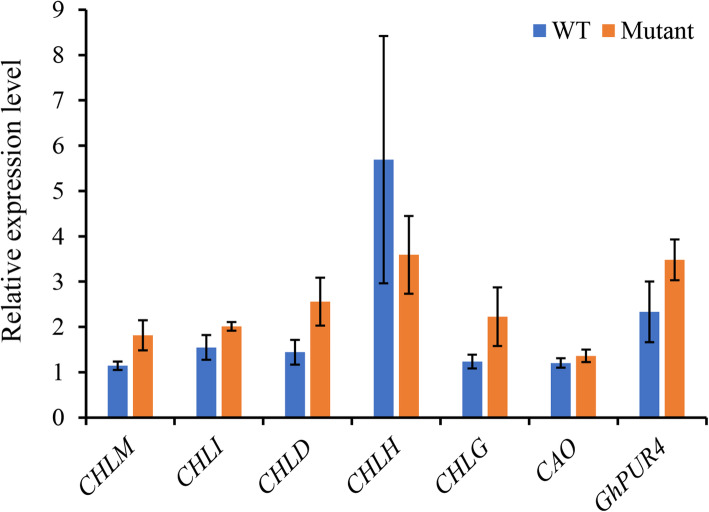
Relative levels of genes in chlorophyll synthesis via qPCR experiments (mean ± SD). *Significantly different between wild type (WT) and virescent mutant of Sumian 22 cotton (*P* < 0.05)

Seven genes in relation to chlorophyll synthesis were selected to for RT-qPCR. The results showed no significant differences between mutant and WT (Fig. 3), suggesting that the reported mechanism of *GhRVL* mutation (identical to CHLI in the present study) [11, 12] in virescent T582 cotton was not responsible for the virescent mutation in Sumian 22.

**Fig. 4:**
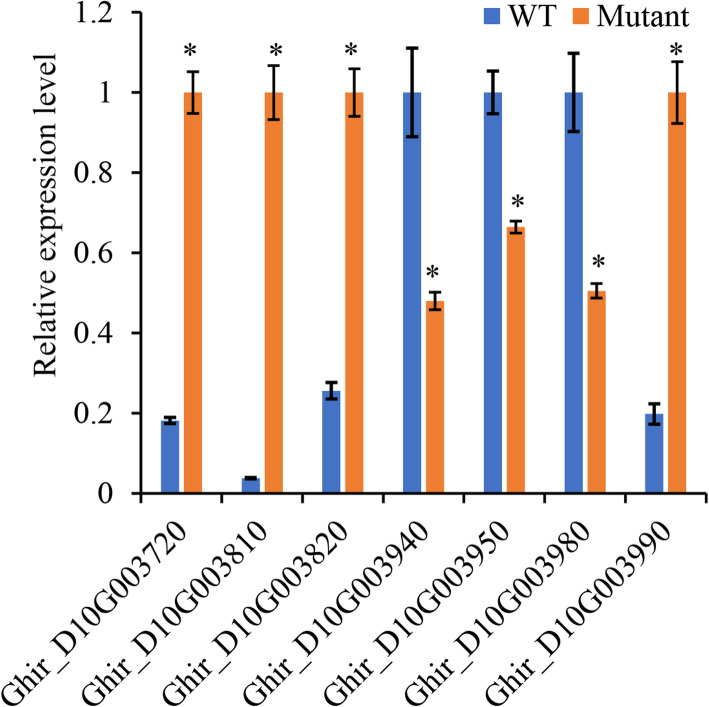
Relative levels of genes in the candidate mutated region in virescent mutant (Mutant) compared with wild type (WT) of Sumian 22 cottons via qPCR experiments (mean ± SD). *Significantly different between WT and Mutant (*P* < 0.01). Ghir_D10G003720: Protein SRG1 (SRG1); Ghir_D10G003810: Floral homeotic protein AGAMOUS (AG); Ghir_D10G003820: Integrin-linked protein kinase 1 (ILK1); Ghir_D10G003940: ER lumen protein-retaining receptor A (ERD2A); Ghir_D10G003950: Protein gravitropic in the light 1 (GIL1); Ghir_D10G003980: ABC transporter I family member 1 (ABCI1); Ghir_D10G003990: unknown

To compare the levels of the 31 genes in the candidate region, RT-qPCR assays were performed for both mutant and WT. Among them, 4 genes (Protein SRG1 (SRG1), Floral homeotic protein AGAMOUS (AG), Integrin-linked protein kinase 1 (ILK1), and a function unknown gene) revealed significantly higher, but 3 genes (ER lumen protein-retaining receptor A (ERD2A), Protein gravitropic in the light 1 (GIL1), and ABC transporter I family member 1 (ABCI1)) displayed significantly lower levels in mutant than in WT (Fig. 4). As reported, these genes were mainly functionally related to plant immunity [25, 26], carpels development [27], and signal transducer activity [28]. Only ABCI1 (Ghir_D10G003980) was reported to participate in the chlorophyll biosynthesis process [29], thus was considered as the candidate gene for virescent mutation. As reported, ABCI1 was suspected to involve in the Proto-IX transport and distribution in *Arabidopsis*, which is an essential step of chlorophyll biosynthesis [30].

### Contents of chlorophyll precursors and chlorophylls

**Fig. 5:**
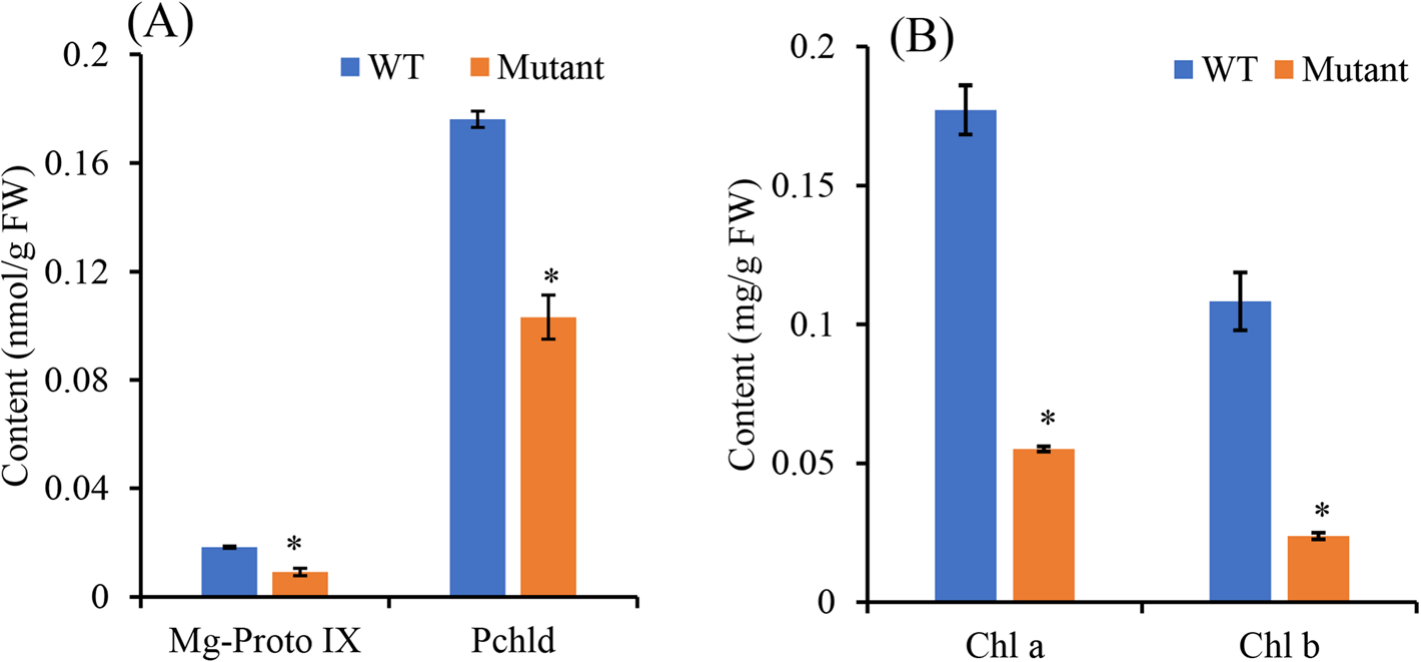
Contents of chlorophyll precursors (**A**) and chlorophylls (**B**) in virescent mutant (Mutant) compared with wild type (WT) of Sumian 22 cottons (mean ± SD). *Significantly different between WT and Mutant (*P* < 0.01)

To validate the metabolic changes of ABCI1 mutation, intermediate products in the biosynthesis process from protoporphyrin IX (Porto-IX) to chlorophyll were determined. The results showed that contents of Mg-protoporphyrin IX (Mg-Proto IX) and protochlorophyllide (Pchld) were both significantly lower in mutant than in WT (Fig. 5A). Meanwhile, contents of both chlorophyll a and b showed significantly lower values in mutant than in WT (Fig. 5B).

### Characterization of mutations potentially affecting ABCI1 transcription in Sumian 22

The open reading frame (ORF) of ABCI1 in Sumian 22 was 690 bp in length, encoding 229 amino acids. Based on the genome sequencing results, nine SNPs which might potentially affect the transcription of ABCI1 were identified (Additional file 1: Table S2). Among them, one was localized to the exonic region of ABCI1, which is heterozygous in the Y-pool. One was localized to the 5′-untranslated region (5′-UTR). It is heterozygous in the G-pool, but homozygous in the Y-pool. This mutation was in an AT-rich region (Additional file 1: Figure S1). Other seven SNPs were all upstream to both Ghir_D10G003980 and Ghir_D10G003990.

## Discussion

In cotton strains, two virescent mutations have been clarified, which were attributed to the dysfunction of *GhRVL* and *GhPUR4* [1, 12, 14]. However, in the present study, qPCR analyses did observe significant differences in these two genes between mutant and WT, indicating that these two genes were not the mechanisms underlying virescent mutation in Sumian 22 cotton. Thus, WT and virescent mutant of Sumian 22 cotton provide good materials to elucidate novel mechanisms underlying virescent mutation in cotton.

**Fig. 6:**
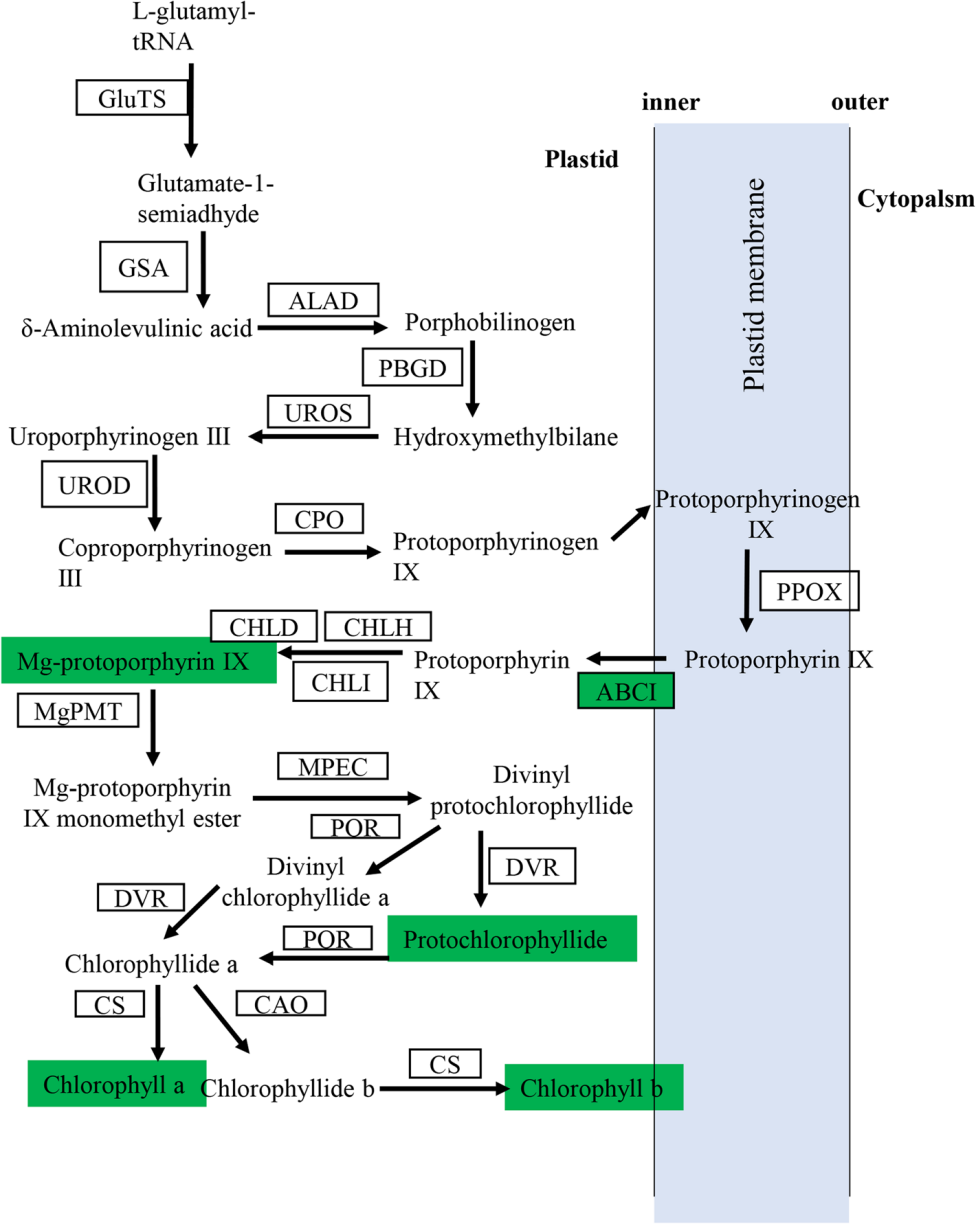
Overview of changes in mRNA transcription and metabolites in the biosynthesis of chlorophyll. Red and green indicate significant upregulation and downregulation in Mutant, respectively, compared with WT

Yellow leaf mutants are often associated with disruptions of photosynthetic pathway, including the regulatory network of chlorophyll biosynthesis and chloroplast development genes. Comparative genome sequencing of WT and mutant only identified one candidate mutation region, which contained 31 genes. In these genes, only ABCI1 was significantly downregulated in mutant in comparison to WT, and functionally related to chlorophyll synthesis. ABC transporter proteins belong to a large, diverse and ubiquitous superfamily [31]. Plants contain a collection of ABC proteins, which are similar to the components of prokaryotic multi-subunit ABC transporters, namely ABC group I. In *Arabidopsis* and rice, ABCI is required for proper formation of chloroplast structure, and biosynthesis of chlorophyll precursor. In addition, ABCI has been characterized as a FR light-specific signaling factor involved in the phytochrome A signaling pathway [30, 32]. During the chlorophyll biosynthesis, protoporphyrinogen IX is relocated from the stroma to the plastid envelope, where it is oxidized by the envelope-bound PPO to form protoporphyrin IX [33]. The generated proto IX then returns to the stroma by yet an unknown mechanism, participating in the synthesis of Mg-proto IX and downstream substances [34]. ABCI1 possibly transfers proto-IX formed on the plastid envelope into the stroma for chlorophyll biosynthesis [30]. Thus, deceased ABCI1 level would negatively affect the amount of proto IX in plastid and subsequently decrease the amounts of Mg-proto IX, Pchld and finally chlorophylls in leaves. Experimental determination really showed significantly lower contents of Mg-proto IX and Pchld (Fig. 5), further supporting that ABCI1 might be a mechanism underlying virescent mutation in Sumian 22 (Fig. 6).

Based on the genome sequences, regulatory mechanisms of ABCI1 transcription were further explored. Nine SNPs were observed in ABCI1 sequence and its upstream 1000 bp region. Among them, the mutations C3329472G and T3330502C could be excluded from the potential regulatory mechanisms of ABCI1, because these two mutations were heterozygous in mutant (Additional file 1: Table S3), which was contradicted to the results of cross-breed experiments that the virescent mutation was recessive in Sumian 22. The mutations G3330282C, C3330324T, A3330374T, T3330536C, C3330589A, and C3330981T might also be excluded, because these mutations were located upstream to both Ghir_D10G003980 (ABCI1) and Ghir_D10G003990 (Additional file 1: Table S3). Thus, these mutations should regulate the transcription of both genes. However, qPCR revealed different changing tendencies between mutant and WT (Fig. 4). Finally, only the mutation A3330215T was left. This mutation was located at an AT-rich region of the 5’-UTR of ABCI1 and is  − 317 bp from the start codon of ABCI1. As reported, a long AT-rich region between positions from − 350 to − 161 bp relative to the transcription start site could function as a cryptic enhancer element regulating the transcription of the following gene in transgenic *Arabidopsis* plants [35]. More investigations are required to validate the regulatory functions of A3330215T mutation on ABCI1 transcription in cotton.

## Conclusions

Virescent mutation in Sumian 22 cotton is recessive. Compared with WT, transcription of ABCI1 was lowered in mutant, inhibiting the transportation of protoporphyrin IX (proto IX) to plastid, and then suppressing the synthesis of Mg-protoporphyrin IX (Mg-proto IX), Protochlorophyllide (Pchld) and finally chlorophylls. These molecular changes are the mechanisms underlying the virescent trait in Sumian 22. The mutation from A to T at the -317 bp position might be the reason to mediate ABCI1 transcription in Sumian 22.

## Materials and methods

### Plant materials and cross-breeding

No special permissions and/or licenses were required for the present study. The collection of all plant materials complied with all current Chinese laws and regulations. The seeds of mutant and WT of Sumian 22 were provided by the Agricultural Sciences Research Institute of Coastal Region of Jiangsu Province (Yancheng, China). Mutant was crossed with WT, and the obtained F_1_ plants were self-pollinated to produce an F_2_ population. Finally, 967 F_2_ seedlings were obtained. These operations were performed at the Nanyang Experimental Station of the Agricultural Sciences Research Institute of Coastal Region of Jiangsu Province (Yancheng, China). The numbers of green and yellow plants were counted to calculated the segregation ratio, and the data were analyzed using the χ^2^ test. In addition, fresh green and yellow leaves were collected and stored at − 80 °C for further molecular analyses.

### Whole genome re-sequencing, and BSA-seq analysis

Leaves from WT, Mutant were extracted using a Biospin plant genomic DNA extraction kit (Bioer, Hangzhou, China). In addition, green leaves (G-pool) and yellow leaves (Y-pool) from the F_2_ population were pooled separately and then subjected to DNA extraction. For each sample, leaves from at least 40 individuals were mixed. DNA degradation and contamination were monitored on 1% agarose gels. DNA purity was checked using a NanoPhotometer spectrophotometer (IMPLEN, CA, USA). DNA concentration was measured using a Qubit DNA assay kit on a Qubit 2.0 Fluorometer (Life Technologies, CA, USA). Sequencing libraries were prepared using a NEBNext Ultra DNA Library Prep Kit for Illumina following the manufacturer’s instruction and subjected to the whole genome sequencing (WGS) using an Illumina HiSeq2500 platform. Paired-end reads were collected. After filtering low-quality reads, the obtained clean data of the two parents and two pools of F2 population were aligned to the reference genome of *G. hirsutum* (AD1) [35] using Burrows-Wheeler Aligner (BWA) (0.7.10-r789) with the default parameters [36]. Alignment/Map (SAM) tools were used to sort and index the resulted Binary Alignment Map (BAM) format files [37]. Mark duplicates in Picard tools (v1.102) (http://broadinstitute.github.io/picard/) was used to discard duplicates, and the final sorted bam results were used for downstream analysis. For all samples, SNP calling was conducted using the Unified Genotype function of the Genome Analysis ToolKit (GATK) (v 3.6) software [38], and then variant calling was performed for SNP and small InDels between bulks.

To identify candidate genomic regions responsible for virescent gene, the SNP-index between the G-pool and Y-pool was estimated as a proportion of reads aligned to a position with a variant nucleotide different from the reference sequence. In order to improve the accuracy of the identified candidate regions, consecutive low depth SNPs were classified into a block with a minimum read depth of 20. A sliding window of 1 Mb long and 10 kb step size was used to measure the average distribution of all SNP-indices. The ΔSNP-index was calculated by subtracting the SNP-index of the G-pool from the Y-pool. Genomic regions with ΔSNP-index higher than the threshold line were considered as candidate regions. Sequences of genes in the candidate regions were extracted, blasted against Nr, Swissport databases for annotation. Alignment of gene and protein sequences were performed using the ClustalX v2.1 software.

### RNA extraction and RT-qPCR

Total RNA was extracted from young leaves of mutant and WT plants using a Plant RNA extraction kit (Bioer, Hangzhou, China). Their quality and quantity were examined using an Agilent Bioanalyzer 2100 (Agilent, USA) and a Qubit RNA assay kit on a Qubit 2.0 Fluorometer (Life Technologies, CA, USA). Then, total RNA were reversed to cDNA using a Prime Script II 1^st^ strand cDNA synthesis kit (Baosheng Bioengineering Institute, Dalian, China). RT-qPCR assays were conducted using SYBR Premix Ex Taq (Baosheng, Dalian, China) on a Gene9600 Plus RT-qPCR machine (Bioer, Hangzhou, China). In total, 31 genes in the candidate regions of the BSA results and seven genes related to chlorophyll biosynthesis were selected for RT-qPCR. The gene names and primers are listed in Additional file 1: Table S3. The cotton actin gene was used as the internal reference. Transcription levels of each gene were compared by calculating their relative change folds using the 2^−ΔΔCt^ method [39]. For each stain, three biological replicates were included.

### Measurement of chlorophyll contents

The contents of chlorophyll a and b (chl-a and chl-b) were measured for WT and mutant leaves [40]. Briefly, fresh leaf samples (0.03 g) were homogenized in 1 ml of extraction solution (acetone:absolute ethanol = 1:1) and then extracted for 18 h in darkness. After centrifugation at 13,000*g* for 5 min, the absorbance values at 645 and 663 nm were measured using a UV4800 spectrophotometer (Unico, Shanghai, China), with the extraction buffer as the blank control. Each treatment was assayed with three biological replicates. Contents of chl-a and chl-b were calculated using the following equations.

$${\text{Chl}} {-} {\text{a }} = {\text{ }}\left( {{\text{12}}.{\text{7A}}_{{{\text{663}}}} - {\text{ 2}}.{\text{69A}}_{{{\text{645}}}} } \right){\text{ }} \times {\text{ V}}/\left( {{\text{1}}000{\text{ }} \times {\text{ W}}} \right)$$

$${\text{Chl}} {-} {\text{b }} = {\text{ }}\left( {{\text{22}}.{\text{9A}}_{{{\text{645}}}} {-}{\text{ 4}}.{\text{68A}}_{{{\text{663}}}} } \right){\text{ }} \times {\text{ V}}/\left( {{\text{1}}000{\text{ }} \times {\text{ W}}} \right)$$

where, A_645_ and A_663_ represent the absorbance at 645 and 663 nm, respectively. W indicates the sample weight (g).

### Measurement of contents of chlorophyll synthesis precursors

To validate the predicted changes of metabolites based on the qPCR results, contents of Mg-proto IX and Pchld were measured using the Hodgins’s method [41]. Briefly, fresh leaf samples (0.05 g) were homogenized in 1 ml of extraction solution (acetone:ammonia = 9:1), and then centrifuged at 13,000*g* for 10 min. The absorbances were measured at 575, 590 and 628 nm. Next, the Mg-proto IX and Pchld contents were calculated using the following equations.

$${\text{Mg}}-{\text{proto IX }} = {\text{ }}0.0{\text{6}}0{\text{77 }} \times {\text{ A}}_{{{\text{59}}0}} {-}{\text{ }}0.0{\text{1937}} \times {\text{ A}}_{{{\text{575}}}} {-}{\text{ }}0.00{\text{3423 }} \times {\text{ A}}_{{{\text{628}}}}$$

$${\text{Pchld }} = {\text{ }}0.0{\text{3563 }} \times {\text{ A}}_{{{\text{628}}}} + {\text{ }}0.00{\text{7225 }} \times {\text{ A}}_{{{\text{59}}0}} {-}{\text{ }}0.0{\text{2955 }} \times {\text{ A}}_{{{\text{575}}}}$$

where A_575_, A_590_ and A_628_ indicate the absorbance at 575, 590 and 628 nm, respectively.

## Statistical analysis

Difference in all indices between WT and Mutant were analyzed by the Student’s *t*-test. Values were considered significantly different with the threshold of *P* < 0.05.

## Supplementary Information

**Additional file 1.** Additional results. Additional figures and tables in the docx format. **Figure S1.** Sequences of GhABCI1 in virescent mutant and wild type. The mutation at 3330215 from T to A is labeled in white back ground. Start and stop codons are labeled in black frame. **Table S1.** Summary of genome sequencing data. **Table S2.** The SNPs potentially affecting Ghir-D10G003980 transcription. **Table S3.** Oligonucleotide primers used in the present study.

## Data Availability

The raw data of whole sequencing have been deposited in the NCBI database with the access number of PRJNA733888 (https://www.ncbi.nlm.nih.gov/bioproject/PRJNA733888).

## Abbreviations

ABCI1: ABC transporter I family member 1
BP: Biological processes
BSA: Bulked-segregant analysis
CC: Cellular components
CHLI: Mg-chelatase I subunit
SNP: Single nucleotide polymorphisms
Mutant: Virescent mutant
WT: Wild green type
