# Supplementary material for: Genome sequencing identified novel mechanisms underlying virescent mutation in upland cotton Gossypiuma hirsutum
Source: BMC Genomics. 2021 Jul 3;22:498. doi: 10.1186/s12864-021-07810-z (PMC8254239; doi:10.1186/s12864-021-07810-z)
Supplement: Supplementary file 1 — Additional file 1. Additional results. Additional figures and tables in the docx format. Figure S1. Sequences of GhABCI1 in virescent mutant and wild type. The mutation at 3330215 from T to A is labeled in white back ground. Start and stop codons are labeled in black frame. Table S1. Summary of genome sequencing data. Table S2. The SNPs potentially affecting Ghir-D10G003980 transcription. Table S3. Oligonucleotide primers used in the present study. [file 12864_2021_7810_MOESM1_ESM.docx]

**Supporting information:**

**Genome sequencing identified novel mechanisms underlying virescent mutation in upland cotton *Gossypiuma hirsutum***

Jin Gao^1^, Yang Shi^1^, Wei Wang^1^, Yong-Hui Wang^1^, Hua Yang^1^, Qing-Hua Shi^1^, Jian-Ping Chen^1^, Yan-Ru Sun^1^, Li-Wang Cai^1*^

^1^ *Institute of Agricultural Sciences of Jiangsu Coastal Area/Observation and Experimental Station of Saline Land of Costal Area, Ministry of Agriculture, Yancheng City, Jiangsu Province, 224002, P. R. China*

*Corresponding author:

Li-Wang Cai, E-mail: jsclw86@163.com, Tel: +86-13961930603

**
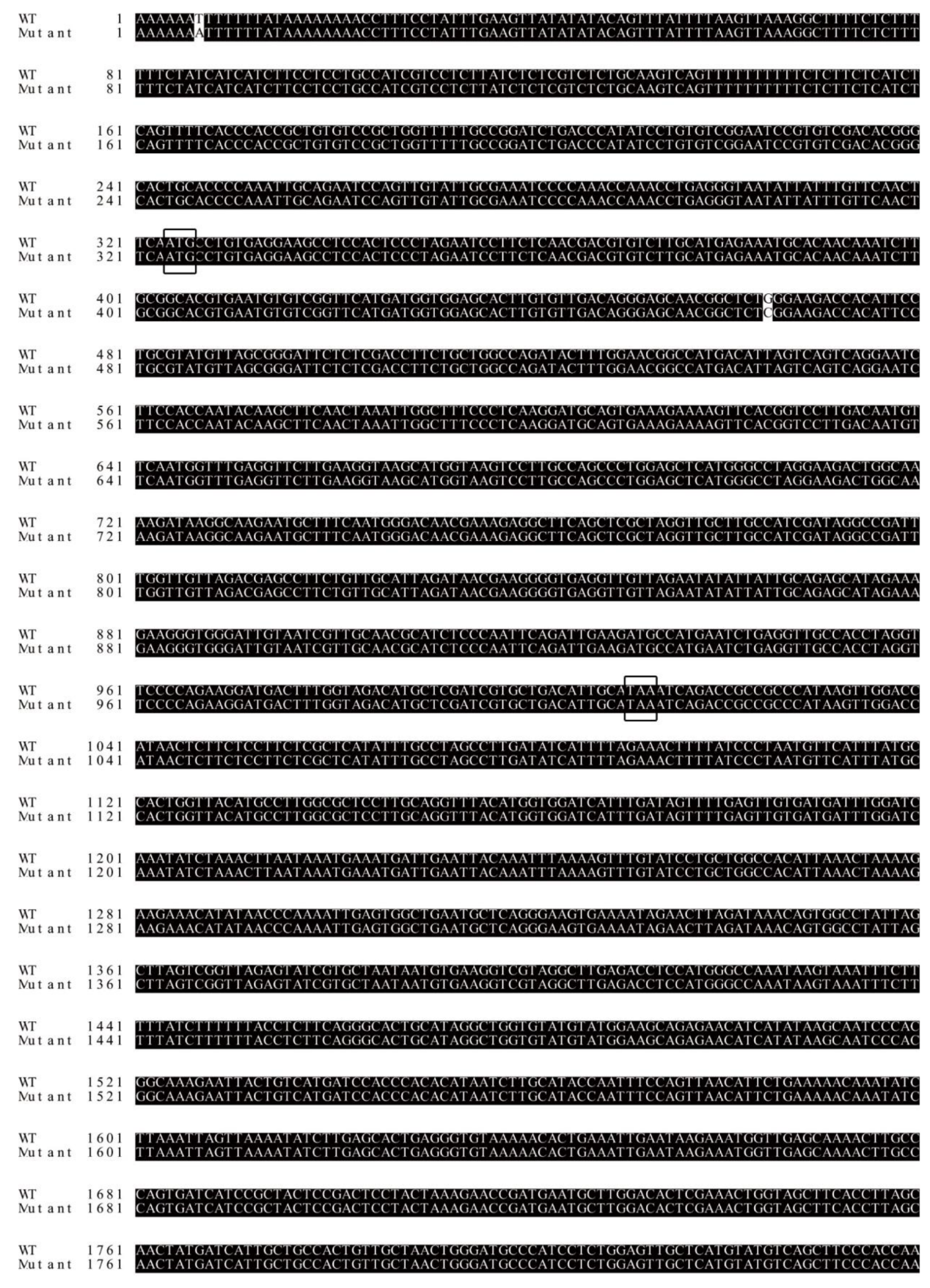
**

**Figure S1. Sequences of GhABCI1 in virescent mutant and wild type.** The mutation at 3330215 from T to A is labeled in white back ground. Start and stop codons are labeled in black frame.

**Table S1. Summary of genome sequencing data.**

| Sample | Total reads | Mapping rate (%) | Average depth (×) | Genome coverage (%) |
| --- | --- | --- | --- | --- |
| WT | 248,925,568 | 98.68 | 13 | 94.66 |
| Mutant | 255,334,194 | 99.30 | 10 | 94.63 |
| G-pool | 822,925,750 | 97.48 | 42 | 95.30 |
| Y-pool | 680,679,302 | 98.66 | 35 | 95.30 |

**Table S2. The SNPs potentially affecting Ghir-D10G003980 transcription.**

| Chromosome | Position | WT | Mutant | Y-pool | G-pool | Location to affecting genes | Affecting genes |
| --- | --- | --- | --- | --- | --- | --- | --- |
| Ghir_D10 | 3329472 | C | G | S | C | exonic | Ghir_D10G003980 |
| Ghir_D10 | 3330215 | A | T | T | W | UTR5 | Ghir_D10G003980 |
| Ghir_D10 | 3330282 | G | C | C | S | upstream | Ghir_D10G003980, Ghir_D10G003990 |
| Ghir_D10 | 3330324 | C | T | T | Y | upstream | Ghir_D10G003980, Ghir_D10G003990 |
| Ghir_D10 | 3330374 | A | T | T | W | upstream | Ghir_D10G003980, Ghir_D10G003990 |
| Ghir_D10 | 3330502 | T | C | Y | Y | upstream | Ghir_D10G003980, Ghir_D10G003990 |
| Ghir_D10 | 3330536 | T | C | C | Y | upstream | Ghir_D10G003980, Ghir_D10G003990 |
| Ghir_D10 | 3330589 | C | A | A | M | upstream | Ghir_D10G003980, Ghir_D10G003990 |
| Ghir_D10 | 3330981 | C | T | T | Y | upstream | Ghir_D10G003980, Ghir_D10G003990,  Ghir_D10G004000 |

**Table S3. Oligonucleotide primers used in the present study.**

| Gene description | Primer sequence (5' to 3') | Fragment size (bp) |
| --- | --- | --- |
| 3720-F | CTTAGCCTTATCGGTCCA | 95 |
| 3720-R | TTGCCATCTAGTTTCCTTG |  |
| 3730-F | GAAAAGGAAATGATAGAAT | 275 |
| 3730-R | AACAAAAGAGAGAGAGATG |  |
| 3810-F | TCACATTGTTGTTGCGGTT | 280 |
| 3810-R | ATAATTGGTGGCGGGTTG |  |
| 3820-F | GAGAGAGGTAAGGATGGAT | 221 |
| 3820-R | TGAAGTAGAAAGGAGACGA |  |
| 3830-F | TAGAGGTTTGCACGAAGG | 124 |
| 3830-R | TGAGTCAAGGATGAGGGAT |  |
| 3840-F | TCCTCCTTCATCAGCTCCA | 220 |
| 3840-R | TACATTCACCACCACCGC |  |
| 3860-F | GCAATCCCCATAAAGCGA | 166 |
| 3860-R | ACAATGATGTCCGAGCCG |  |
| 3870-F | GTTTTCACGGCGGGGCTC | 214 |
| 3870-R | AGGGACGGCGTTCTCTGGTT |  |
| 3880-F | CCTGCTGCTCGCCATTAT | 182 |
| 3880-R | GCGGGTGCTTGAGACATT |  |
| 3890-F | CGCAGGTGGTTCCATAAGT | 251 |
| 3890-R | TTCGGGAGACAAGGCTAAA |  |
| 3900-F | TGTATGGCTGAGGGTTTA | 236 |
| 3900-R | AAGATGGAAGAGGAGAGG |  |
| 3910-F | GTGAAATTCGTTTGCTGG | 78 |
| 3910-R | GTAAGTGTGGTATCGCCC |  |
| 3920-F | CGTCTTTGTGTGCCTTGA | 214 |
| 3920-R | TCCGCCTTAGATTTGGTC |  |
| 3930-F | ACCGATTACGAGGACACAAC | 244 |
| 3930-F | CCGACTCTCATTTCCAGGA |  |
| 3940-R | ATTTCATCAGCCTTGCCTAT | 104 |
| 3940-F | CCATTCATTTTTCACCTTCC |  |
| 3950-F | TGATGTTATTCACGCTCG | 204 |
| 3950-R | GTTGCTCCTCCATTTTCT |  |
| 3960-F | GCAATGCAATTTCTAGTG | 206 |
| 3960-R | TAAGTGATGGAGGGTGTT |  |
| 3970-F | TGAGGAGGAAGAAAGAAG | 234 |
| 3970-R | CATACAGGGGAAAAAGTG |  |
| 3980-F | CTTGTGTTGACAGGGAGC | 162 |
| 3980-R | GAGGGAAAGCCAATTTAG |  |
| 3990-F | ACCTATTTACACCACCAG | 230 |
| 3990-R | TGAACTCACAGTACTCCC |  |
| 4000-F | TACATTCCCAACCCACCAA | 264 |
| 4000-R | TTAGACTTCCCCACACCTGA |  |
| 4010-F | TAACAGTGAGGCGGATGAC | 108 |
| 4010-R | TGCCGAAACACAAACGAG |  |
| 4020-F | GTCAAGACCAGAAAACAAG | 268 |
| 4020-R | CAAAAACGAACATCACAC |  |
| 4030-F | GAATGAGATGATTGTGTG | 130 |
| 4030-R | ATATCCGTATGTGGAGTT |  |
| 4040-F | AGGACCGAGTGGAGGGAT | 77 |
| 4040-R | CACGGATAAAACAGAGGGG |  |
| 4050-F | GCCATTTGCCTGTTCCTAT | 177 |
| 4050-R | AACCACCTTCTAATCCCCC |  |
| 4060-F | GCCCATCTCTTTGCCGTC | 229 |
| 4060-R | GTGGTCCCCAAGCTTCGTA |  |
| 4070-F | TATGTCACCTCCTCCTTCT | 88 |
| 4070-R | GTTTTCACTTGTATCGTCTG |  |
| 4090-F | CGAGTGCAAAGATGAGCT | 134 |
| 4090-R | TATTTTCCCCGAAGAGAC |  |
| 4100-F | GTCCTACTCCGAAAATAC | 262 |
| 4100-R | AGACATCAACAACATACCA |  |
| CHLM-F | TGTTTGCGATGCTGGTTGTG | 130 |
| CHLM-R | TAACTGCTCTTTCGCCTGCT |  |
| CHLI-F | ATAACTCGGACCCGGAGGAT | 193 |
| CHLI-R | GGCTCAAATGCCTTGACACC |  |
| CHLD-F | AAAGTTGCGTCGGGAGAGAG | 171 |
| CHLD-R | AGTGCAGCTCCTTTAGCGTT |  |
| CHLH-F | TATGCCTGGAAAGCAGGTGG | 169 |
| CHLH-R | GTTTTCAGCCGGAGGAGTCA |  |
| CHLG-F | CCTGTAGCCTTTGGTGCTGA | 119 |
| CHLG-R | AGGGCGAGTGCATAATACGG |  |
| CAO-F | ATGAAGAGGCCCCGGAAAAG | 183 |
| CAO-R | GCATCCTGGCTTCCCATCTT |  |
| GhPUR4-F | GCTCTTCTCGGTCAAAGCC | 155 |
| GhPUR4-R | GAGAAGCTCGTCGTTTGCAC |  |
| actin-F | GACCGCATGAGCAAGGAGAT | 130 |
| actin-R | GCTGGAAGGTGCTGAGTGAT |  |
